# Retraction: Pathological Roles of Interleukin-22 in the Development of Recurrent Hepatitis C after Liver Transplantation

**DOI:** 10.1371/journal.pone.0225971

**Published:** 2019-11-26

**Authors:** The PLOS ONE Editors

Concerns have been raised that the transplants performed in the local context of procedures reported in this article [1] may have involved organs/tissues procured from prisoners [2]. International ethics standards call for transparency in organ donor and transplantation programs and clear informed consent procedures including considerations to ensure that donors are not subject to coercion.

Details as to the transplant donor sources and methods of obtaining informed consent from donors were not reported in [1]. The authors did not clarify these issues or the cause(s) of donor death in response to journal queries, although they provided ethics approval documentation and informed consent forms for organ donors. The first author claimed that the authors were not involved with organ donation or surgical procedures and noted that this study focused on transplant recipients. The Ethics Statement of [1] declared that patients provided informed written consent, and stated, “No conflict of interest during all organ transplantations. No organ trafficking involved.” The ethics approval document provided (S1 Fig in [1]) was dated after the onset of participant recruitment and it indicated that the ethics review and approval did not include consideration of an informed consent form. Furthermore, while the ethics document indicated that no organ trafficking was involved it did not clarify donor sources or whether organs had been procured from prisoners.

The authors did not provide the primary data underlying this article’s results and as such the article does not comply with the PLOS Data Availability Policy.

Owing to the above concerns, and in line with international ethics standards for organ/tissue donation and transplantation, the *PLOS ONE* Editors retract this article.

YG did not agree with retraction. HR, FM, JL, EC, HL, JZ, HL, ZL, and MZ did not respond or could not be reached.
